# Novel Antiplatelet Agent Use for Acute Coronary Syndrome in the Emergency Department: A Review

**DOI:** 10.1155/2013/127270

**Published:** 2013-02-21

**Authors:** M. Curial, E. Nath, E. Lang

**Affiliations:** Department of Family Medicine, University of Calgary, 1632 14 Avenue NW, Calgary, AB, Canada T2N 1M7

## Abstract

*Background*. Acute Coronary Syndrome (ACS) is a clinical condition encompassing ST Segment Elevation Myocardial Infarction (STEMI), Non-ST Segment Elevation Myocardial Infarction (NSTEMI), and Unstable Angina (UA) and is characterized by ruptured coronary plaque, ischemic stress, and/or myocardial injury. Emergency department (ED) physicians are on the front lines of ACS management. The role of new antiplatelet agents ticagrelor and prasugrel in acute ED management of ACS has not yet been defined. *Objective*. To critically review clinical trials using ticagrelor and prasugrel in the treatment of ACS and inform practitioners of their potential utility in treating ACS in the ED. *Results*. Trials on the efficacy of ticagrelor and prasugrel achieve statistical significance in decreasing composite endpoints in select patient populations. *Conclusion*. The use of ticagrelor and prasugrel as first line ED treatment of ACS is not well established. Current evidence supports the use of several agents with the final decision based on treatment protocols conjointly developed between cardiology and emergency medicine (EM). Further clinical trials involving head-to-head trials or comparisons of drug-based strategies are required to show superiority in reducing cardiac endpoints with regard to ED initiation of treatment.

## 1. Introduction

Acute Coronary Syndrome (ACS) is a clinical syndrome comprising ST Segment Elevation Myocardial Infarction (STEMI), Non-ST Segment Elevation Myocardial Infarction (NSTEMI), and Unstable Angina (UA). ACS is a common and important diagnosis that is often made in the emergency department by front-line physicians and rapid recognition and diagnosis of ACS, risk stratification, and appropriate treatment have been shown to decrease morbidity and mortality [1].

In the context of ACS treatment, dual antiplatelet therapy with ASA and clopidogrel (a P2Y12 receptor inhibitor) reduces rates of harmful cardiac events such as cardiovascular causes of death, myocardial infarction, and stroke [2]. However, with new agents undergoing evaluation in large clinical trials, acute care providers need to know if the new P2Y12 receptor inhibitor antiplatelet agents ticagrelor and prasugrel are clinically superior to the current standard clopidogrel.

It has been established that a defined percentage of the population exhibits high platelet activity despite the use of clopidogrel. This phenomenon occurs anywhere from 5% to 44% of patients studied depending on the clopidogrel dose and patient population [3]. It is uncertain what level of platelet activity during ACS is related to harmful cardiovascular outcomes such as cardiovascular death, myocardial infarction, and stroke [4]. Ticagrelor and prasugrel have been demonstrated to reduce levels of platelet activation when compared to clopidogrel [5, 6] which could lead to reduced risk of thrombosis and improved artery or stent patency. However, there is debate as to which patients will gain the most clinical benefit from these costly and potentially harmful agents.

In this paper we will critically appraise the few large clinical trials that examine the use of ticagrelor or prasugrel in the treatment of ACS and highlight current treatment guidelines for ACS. Our focus will be on information relevant to EM and other acute care physicians.

## 2. The Novel Antiplatelet Agents

Clopidogrel, prasugrel, and ticagrelor are all examples of P2Y12 receptor inhibitor antiplatelet medications that can be used in the treatment of ACS. Clopidogrel and prasugrel both are irreversible inhibitors of the P2Y12 receptors on the platelet surface. Platelets inhibited by these two agents are affected for the remainder of their lifespan, and therefore platelet aggregation returns to baseline within 5–10 days of discontinuation of either drug. Clopidogrel is a prodrug that is activated in the liver by cytochrome P450 enzymes and genetic variability in enzyme function is known to cause the medication to be less effective in individuals who cannot convert the drug to its active form. Prasugrel is a prodrug as well but appears to be effective in most individuals. In contrast ticagrelor is a reversible noncompetitive antagonist of the P2Y12 receptor; its action and the recovery of platelet function likely depend on the serum concentration of the drug. Ticagrelor is not a prodrug.

Of the three agents, clopidogrel has the longest onset of action at 2 hours after administration of the initial loading dose. Both ticagrelor and prasugrel cause inhibition of platelet activity (IPA) within 30 minutes of the initial loading dose, and their time to achieve maximal IPA is 4–8 hours [7]. Clopidogrel has the shortest half-life elimination of its active metabolite at 30 minutes. The half-life of ticagrelor and prasugrel is 9 and 7 hours on average, respectively.

Both ticagrelor and prasugrel show increased risk of bleeding. Additionally ticagrelor can cause dyspnea as an adverse effect in 10–14% of patients. Dyspnea usually occurs early in the course of treatment and is self-limited. Ticagrelor is contraindicated for use in patients who are taking medications that are strong CYP3A4 inhibitors; it is also contraindicated in those patients who have a history of intracranial hemorrhage. Prasugrel is contraindicated in patients with a history of transient ischemic attack (TIA) or stroke.  Table 1 compares and contrasts these agents.

## 3. Prasugrel

Data on the efficacy of prasugrel in the treatment of ACS comes from two large, industry-sponsored clinical trials: TRITON-TIMI 38 [11] and TRILOGY ACS [12].

### 3.1. TRITON-TIMI 38

The major study comparing prasugrel to clopidogrel is TRITON-TIMI 38. It compared prasugrel (60 mg loading dose, 10 mg daily maintenance dose) to clopidogrel (300 mg loading dose, 75 mg daily maintenance dose) in a double-blind, double-dummy, randomized control clinical trial. The study population was moderate to high risk ACS patients scheduled for PCI; 13,604 patients were enrolled with 10074 representing the UA/NSTEMI range of the ACS spectrum and 3534 with STEMI. Eligibility-defining risk was calculated using the TIMI risk score [13]. NSTEMI/UA patients could be enrolled within 72 hours of symptom onset and STEMI patients could be enrolled within 12 hours after onset of symptoms for patients undergoing primary PCI and within 14 days after onset of symptoms for those managed medically. 99% of patients received PCI. Patients were followed for a minimum of 6 months up to a maximum of 15 months.

The primary endpoint of this study was a composite of (1) death from cardiovascular cause, (2) nonfatal myocardial infarction, and (3) nonfatal stroke measured for an average of 14.5 months after randomization. Results were analyzed in an intention to treat analysis and showed the following:

- decreased rates of primary endpoint in the NSTEMI/UA group treated with prasugrel (hazard ratio, 0.82; 95% confidence interval (CI) 0.73 to 0.93; *P* = 0.002; could not calculate the number needed to treat (NNT) due to absent raw data),
- decreased rates of primary endpoint in STEMI group treated with prasugrel (hazard ratio, 0.79; 95% CI 0.65 to 0.97; *P* = 0.02; could not calculate the number NNT due to absent raw data),
- decreased rates of primary endpoints overall for all patients treated with prasugrel (hazard ratio, 0.81; 95% confidence interval, 0.73 to 0.90; *P* < 0.001; NNT 46). This can be further broken down into
  - a nonsignificant decrease in death from cardiovascular causes for all patients treated with prasugrel (hazard ratio, 0.89; 95% confidence interval 0.70–1.12; *P* = 0.31),
  - a significant decrease in nonfatal MI for all patients treated with prasugrel, the primary driver of the composite endpoint (hazard ratio, 0.76 ; 95% confidence interval 0.67 to 0.85; *P* < 0.001; NNT 46),
  - a non-significant increase in nonfatal stroke for all patients treated with prasugrel (hazard ratio, 1.02 ; 95% confidence interval, 0.71–1.45; *P* = 0.93).

The primary safety endpoint of this study was major bleeding as defined by TIMI major bleeding criteria. This showed

- a significant increase in the rate of non-CABG-related major bleeding (hazard ratio, 1.32; 95% CI 1.03–1.68; *P* = 0.03; number needed to harm (NNH) 167) further broken down to
  - a significant increase in the rate of life-threatening bleeding (hazard ratio, 1.52; 95% CI 1.08–2.13; *P* = 0.01 ; NNH 200),
  - a significant increase in the rate of fatal bleeding (hazard ratio, 4.19; 95% CI 1.58–11.11; *P* = 0.002 ; NNH 334),
- a significant increase in the rate of bleeding requiring transfusion (hazard ratio, 1.34; 95% CI 1.11–1.63; *P* < 0.001 ; NNH 100),
- a significant increase in the rate of CABG-related major bleeding (hazard ratio, 4.73; 95% CI 1.90–11.82; *P* < 0.001 ; NNH 10).

Because of the increased risk in bleeding, a post hoc analysis was conducted and found three specific subgroups in which the benefit from prasugrel did not outweigh harm:

- patients with a history of previous stroke or TIA showed statistically significant net harm (hazard ratio, 1.54; 95% CI 1.02–2.32; *P* = 0.04),
- patients 75 years old and older showed no benefit to treatment with prasugrel (hazard ratio, 0.99; 95% CI 0.81–1.21; *P* = 0.92),
- patients under 60 kilograms showed no benefit to treatment with prasugrel (hazard ratio, 1.03; 95% CI 0.69–1.53; *P* = 0.89).

Data from this trial suggests clinical superiority of prasugrel over clopidogrel in preventing the composite cardiac endpoint when used in moderate to high risk patients with planned PCI. This superiority is mainly seen in preventing nonfatal myocardial infarction with little or no impact on rates of cardiac death and nonfatal stroke. For the purpose of this study, nonfatal MI was defined as “distinct from the index event and… defined by symptoms suggestive of ischemia/infarction, electrocardiographic data, cardiac biomarker, or pathologic evidence of infarction dependent on the clinical situation” [14]. 

The study also suggests that treatment with prasugrel results in a small but statistically significant increase in bleeding, especially fatal bleeding. These rates appeared higher in three subgroups: patients with previous stroke or TIA, patients 75 years old or older, and patients weighing less than 60 kg. This information should serve as a caution when selecting patients likely to benefit from prasugrel administration and suggests avoiding this medication in the previously mentioned populations.

Critical appraisal of this study suggests several limitations in determining which antiplatelet agent should be used for the acute ACS patient presenting to the ED. First, the appropriate loading dose of clopidogrel is currently being questioned in the literature with many specialists advocating a larger 600 mg loading dose as opposed to the 300 mg dose used in this study [15–18]. Use of a potentially suboptimal comparator might have biased the outcomes reported.

It is worth noting that patients were administered the study medication at any point between randomization up to 1 hour after leaving the catheterization laboratory. It is not clear how results would change if patients were started on dual antiplatelet therapy at the time of diagnosis (pretreatment). ACCOAST [19] is a current clinical trial investigating the risks and benefits of pretreating patients with 30 mg of prasugrel at the time of ACS diagnosis and 30 mg more at the time of PCI versus 60 mg at the time of PCI only. Results from this trial are expected in early 2013 and will be very relevant to ED physicians.

TRITON-TIMI 38 is only applicable to moderate and high risk patients scheduled for PCI. It is difficult to determine what benefit patients not undergoing PCI would experience in terms of efficacy and bleeding risk. TRILOGY ACS, described below, fills that gap in knowledge.

### 3.2. TRILOGY ACS

TRILOGY ACS is a recent study which examined the effect of prasugrel usage in UA and NSTEMI patients not undergoing revascularization. Patients were randomized in the study only after a decision for medical management without revascularization was made. In addition, patients must have been classified as high risk by possessing at least one of the following characteristics:

1. age of at least 60 years old,
2. presence of diabetes mellitus,
3. previous myocardial infarction,
4. previous revascularization with either PCI or coronary artery bypass grafting (CABG).

Patients were excluded if they had a history of TIA or stroke, PCI or CABG within 30 days, renal failure on dialysis, or concomitant anticoagulant treatment.

This study was designed to assess the efficacy of prasugrel (10 mg daily dose) versus clopidogrel (75 mg daily dose) in long-term maintenance therapy for ACS patients that did not receive revascularization and used the same composite endpoint as TRITON-TIMI 38. Patients were enrolled up to 10 days after treatment decision reducing the applicability of this paper to ED care. 

Results of the primary analysis [12] of patients under 75 years old study show no statistically significant change in the rate of the primary endpoint between clopidogrel and prasugrel groups. 

## 4. Ticagrelor

PLATO is the major study pitting ticagrelor against clopidogrel in ACS patients [20]. This industry-funded multicenter, randomized, double-blind, double-dummy trial compared ticagrelor (180 mg loading dose, 90 mg bid maintenance dose) against clopidogrel (300–600 mg loading dose, 75 mg daily thereafter). The study population included patients admitted to hospital with ACS, with or without ST segment elevation, with an onset of symptoms during the previous 24 hours. 18,624 patients were enrolled in this study with 9333 randomly assigned to ticagrelor and 9291 randomly assigned to the clopidogrel group. 

The primary endpoint of this study was a composite of (1) death from vascular causes, (2) nonfatal myocardial infarction, and (3) nonfatal stroke. For the purpose of this study, non fatal MI was defined as follows:

- recurrent MI within 18 hours of a previous MI—defined as recurrent cardiac ischemic symptoms and a new ST elevation;
- recurrent MI after 18 hours but before cardiac markers have returned to normal—defined as symptoms and reelevation of troponin or CK-MB of at least 50% over a previous value that was decreasing;
- MI after cardiac biomarkers have returned to normal—defined as elevation of biochemical markers above the upper limit of normal with either ischemic symptoms at rest, ECG changes, or pathological findings of an acute MI;
- MI within 24 hours after PCI—defined as cardiac biomarkers ≥3x the local laboratory upper limit of normal from a normal or decreasing level and after CABG ≥10x the upper limit of normal or ≥5x with new Q waves.

ST segment elevation was described as the persistent elevation of the ST segment of 1 mm or more in two or more contiguous leads or a new left bundle branch block and the need for PCI. 

Analyses were by intention to treat and results showed

- a significant decrease in the primary composite endpoint for patients receiving ticagrelor (hazard ratio 0.84; 95% confidence interval 0.77–0.92; *P* < 0.001; NNT = 53). This composite can be further broken down into
  - a significant decrease in death from vascular causes for all patients treated with ticagrelor (hazard ratio, 0.79; 95% CI 0.69–0.91; *P* = 0.001; NNT 91),
  - a significant decrease in nonfatal MI for all patients treated with ticagrelor (hazard ratio, 0.84; 95% CI 0.75–0.95; *P* = 0.005; NNT 91),
  - a nonsignificant increase in nonfatal stroke for all patients treated with ticagrelor (hazard ratio, 1.17; 95% CI 0.91–1.52; *P* = 0.22).

The primary safety endpoint of this study was major bleeding as defined by the TIMI major bleeding criteria and/or a study-specific set of criteria. Overall there were no statistical differences between the two groups with regard to major or life-threatening bleeding. The only statistically significant differences in primary safety endpoints were

- a significantly higher instance of fatal intracranial bleeding in the ticagrelor group (11/9235 (0.1%) versus 1/9186 (0.01%), *P* = 0.02; NNH 112),
- a significantly lower rate of nonintracranial fatal bleeds in the ticagrelor group (9/9235 (0.1%) versus 21/9186 (0.3%), *P* = 0.03; NNT 500).

When closer analyzed, a statistically significant difference in non-CABG-related major bleeding was seen as follows:

- as defined by study criteria (hazard ratio, 1.19; 95% CI 1.02–1.38; *P* = 0.03; NNH 143) and
- as defined by TIMI criteria (hazard ratio, 1.25; 95% CI 1.03–1.53; *P* = 0.03; NNH 167).

Additionally, premature discontinuation of the study drug due to adverse events occurred more frequently with ticagrelor than with clopidogrel (in 7.4% of patients versus 6.0%; *P* < 0.001).

Data from this trial suggests clinical superiority of ticagrelor over clopidogrel in preventing the composite primary endpoint; however, patients on ticagrelor were observed to have higher rates of major bleeding not related to CABG and more instances of fatal intracranial bleeding. Patients in the study also more frequently discontinued ticagrelor due to adverse effects. 

A benefit of this study with regards to its relevance to ED physicians is that all patients were randomized within 24 hours of symptom onset. Additionally, both invasively managed and noninvasively managed patients were enrolled. This early randomization and administration of study drug as well as a broader study population is more applicable to EM management of ACS.

## 5. Prasugrel versus Ticagrelor

Absent from this body of research is a direct comparison of prasugrel to ticagrelor. Since the two medications have never been studied in a head-to-head trial, they are difficult to compare. Recently, an international group attempted to compare the efficacy of prasugrel to that of ticagrelor in an indirect meta-analysis with data on prasugrel coming from the TRITON-TIMI 38 trial and data for ticagrelor from the DISPERSE-2 and PLATO trials (Table 2) [21]. Final conclusions from this study suggest that both prasugrel and ticagrelor are superior to clopidogrel and that these two drugs have similar efficacy and safety profiles.

## 6. Overview of Current Treatment Guidelines for ACS

We feel that treatment of ACS should be based on easy-to-apply protocols to help increase the speed of delivery of treatment and minimize medical errors in complex treatment regimens. Rigorously developed clinical practice guidelines are available from the European Cardiology Society and the American Society of Cardiology. We will focus our analysis on the suggested roles of prasugrel and ticagrelor.

### 6.1. American College of Cardiology/American Heart Association (ACC/AHA) Guidelines

The ACC/AHA created a widely used version of North American based ACS guidelines. Guidelines for STEMI (written in 2004 [22], updated in 2007 [23] and again in 2009 [24]) and for NSTEMI/UA (2007 [25], updated 2011 [26] and 2012 [27]) are in existence.

The initial 2004 guidelines for STEMI recommended initial treatment with ASA as an antiplatelet agent. Additional antiplatelet agents were not recommended in the emergency department. Once diagnostic angiography had been performed, clopidogrel was recommended to be started for patients scheduled to undergo PCI.

In subsequent updates to this document, the role of antiplatelet agents was modified; one primary change focused on the use of thienopyridine antiplatelet agents. The 2009 update recommended the use of clopidogrel as soon as possible in patients that may receive primary or nonprimary PCI (class one recommendation, level of evidence C) or prasugrel as soon as possible for patients that will be receiving primary PCI (class one recommendation, level of evidence B). The guidelines do not address the role of ticagrelor.

The current ACC/AHA NSTEMI guidelines were very recently published in August of 2012 and prasugrel and ticagrelor play a large role in the management of ACS. Dual antiplatelet therapy is now recommended (class one recommendation, level of evidence varies based on presentation and treatment) with ASA and one of clopidogrel, prasugrel, or ticagrelor in both short- and long-term management of ACS. It is important to note that the guideline writing group did not recommend one P2Y12 receptor inhibitor over another.

### 6.2. European Cardiology Society (ESC) Guidelines

The European Cardiology Society produces clinical practice guidelines to help guide medical practitioners in the treatment of ACS. Similar to the ACC/AHA, they have separate documents for NSTEMI/ACS (2011 [28]) and for STEMI/Acute Myocardial Infarction (2012 [29]). 

ECS STEMI guidelines recommend dual antiplatelet therapy with ASA (class 1 recommendation, grade B evidence) and an ADP receptor antagonist (class 1 grade A) as early as possible for patients with planned PCI. The key difference in the ECS guidelines when compared to the ACC/AHA guidelines is that prasugrel and ticagrelor are suggested as the preferred ADP receptor antagonists (class 1 grade B) and suggest clopidogrel only when prasugrel and ticagrelor are contraindicated or unavailable.

The ECS also suggests dual antiplatelet therapy to be started as soon as possible in NSTEMI patients (class 1 grade A). Similar to their STEMI recommendations, the ECS suggests prasugrel or ticagrelor (both class 1 grade B) as the ADP receptor antagonists of choice and again suggests clopidogrel only when prasugrel and ticagrelor are contraindicated or unavailable (class one grade A). Additionally, the ECS guidelines suggest a 600 mg loading dose of clopidogrel for patients scheduled for invasive management when this medication is chosen as the ADP receptor antagonist (class 1 grade B).

## 7. Conclusion

While it has been shown that both prasugrel and ticagrelor can decrease rates of composite cardiac endpoints in carefully selected patients with ACS, the value of initiating treatment with these agents in the ED has not been clarified. Since much of the benefit of these drugs has been shown when given near the time of PCI and in long-term followup after ED discharge, we suggest that whenever possible decisions about antiplatelet agent choice be made in conjunction with the cardiology services providing immediate and long-term care. In general, patients that are at a low risk for bleeding will likely receive the greatest benefit from the new agents. High risk patients with planned PCI will likely benefit from prasugrel. The optimal timing of medication administration has not yet been established.

Having three appropriate agents for use in the current ACS guidelines causes a potential source of human error. With increased choice of therapeutic agents, treatment protocols become less standardized. This opens the door to potential omissions or delayed management as treatment decisions are being made. It is important to remember that current guidelines still focus on early recognition and treatment of ACS including appropriate revascularization and that providing new medications is not the primary goal of ED physicians. 

Current evidence suggests improved efficacy of both prasugrel and ticagrelor over clopidogrel with quicker onset of action. However, these medications also have higher bleeding risks. Initiating treatment with these medications on all patients presenting to the ED with ACS has not been proven to decrease morbidity or mortality and we feel no compelling reason to recommend one agent over another for treatment of ACS in the ED. Instead, we feel the current evidence supports the use of any of these agents with the final decision based on patient presentation, national treatment guidelines, and local institutional treatment protocols. Optimal antiplatelet therapy is a rapidly evolving topic and the ED approach to antiplatelet therapy is likely to evolve in the upcoming months. However, we would like to stress that, despite medication advances, rapid recognition of ACS, risk stratification, and resuscitation when required are still the key roles of the EM physician in managing ACS.

## Figures and Tables

**Table 1:** Pharmacologic properties of P2Y12 receptor inhibitors used in ACS.

	Clopidogrel [8]	Ticagrelor [9]	Prasugrel [10]
Class	Thienopyridine	Nucleoside analogue	Thienopyridine
Prodrug	Yes	No	Yes
Route	Oral	Oral	Oral
Metabolism	Hepatic	Hepatic (CYP34A)	Intestinal, serum, hepatic
Mechanism of action	Active metabolite IRREVERSIBLY inhibits P2Y12 subtype of ADP receptors on the platelet surface, which prevents activation of GIIb/IIIa receptor complex, thereby reducing platelet activation and aggregation; platelets blocked by clopidogrel are affected for the remainder of their lifespan (~7–10 days); note that genetic variability of CYP2C19 may preclude patients from the full effect of drug	REVERSIBLY and noncompetitively binds the P2Y12 subtype of ADP receptors on the platelet surface, which prevents ADP-mediated activation of the GIIb/IIIa receptor complex, thereby reducing platelet aggregation; due to reversible antagonism of the P2Y12 receptor, recovery of platelet function is likely to depend on serum concentrations of drug and its active metabolite	Active metabolite IRREVERSIBLY blocks the P2Y12 subtype of ADP receptors on the platelet, which prevents activation of the GIIb/IIIa receptor complex, thereby reducing platelet activation and aggregation; platelet aggregation returns to baseline within 5–9 days of discontinuation
Onset of action (IPA)	300–600 mg loading dose detected within 2 hours	180 mg loading dose ~41% within 30 minutes	60 mg loading dose within 30 minutes
Time to maximal IPA	6 hours after loading dose	4–8 hours after loading dose	4–8 hours after loading dose
Half-life elimination of active metabolite	~30 minutes	~9 hours	~7 hours (range 2–15 hours)
Excretion	Renal (50%), biliary (46%)	Biliary	Renal (~68%), biliary (~27%)
Significant adverse effects	None	Increased minor/major bleeding	Increased minor/major bleeding
Contraindications	Hypersensitivity, active bleeding, significant liver impairment, and cholestatic jaundice	Hypersensitivity, active bleeding, history of intracranial hemorrhage, hepatic impairment, concomitant use of strong CYP3A4 inhibitors (e.g., ketoconazole, clarithromycin, ritonavir, atazanavir, nefazodone)	Hypersensitivity, active bleeding, history of TIA, or stroke

IPA: inhibition of platelet aggregation.

**Table 2:** Risk of bias assessment of key trials.

	TRITON-TIMI 38		TRILOGY ACS		PLATO	
	Authors judgement	Support for judgement	Authors judgement	Support for judgement	Authors judgement	Support for judgement
Random sequence generation (selection bias)	Unclear risk of bias	No information provided on randomization protocol aside from stratification based on presenting symptoms	Unclear risk of bias	No information provided on randomization protocol aside from stratification by age, country, and prior clopidogrel treatment status	Low risk of bias	Patients randomised using computer software (treating physician could not influence) operating on a randomisation schedule managed by a group not involved in the trial
Allocation concealment (selection bias)	Low risk of bias	Double-dummy trial method used	Low risk of bias	Double-dummy trial method used	Low risk of bias	Double-dummy trial method used
Blinding of participants and personnel (performance bias)	Unclear risk of bias	Trial is “double blinded" but does not refer explicitly who is blinded	Unclear risk of bias	Trial is “double blinded" but does not refer explicitly who is blinded	Unclear risk of bias	Trial is “double blinded" but does not refer explicitly who is blinded
Blinding of outcome assessment (detection bias)	Unclear risk of bias	Trial is “double blinded" but does not refer explicitly who is blinded	Unclear risk of bias	Trial is “double blinded" but does not refer explicitly who is blinded	Unclear risk of bias	Trial is “double blinded" but does not refer explicitly who is blinded
Incomplete outcome data (attrition bias)	Low risk of bias	Intention to treat analysis, only 0.1% of patients lost to followup, raw data for safety and efficacy endpoints provided for overall cohort	Low risk of bias	A similar proportion of the prasugrel and clopidogrel groups (76% versus 78%, resp.) continued to receive the study drug throughout the followup. A small proportion (120 patients from 4 sites) were excluded from analysis due to breaking protocol, this was done prior to unblinding, and therefore the effects were unknown to the authors.	Low risk of bias	Analysis carried out on entire prespecified stratum. Similar proportions of study drug (14.6) and control drug (14.0) treated patients dropped out of the study. Intention to treat analysis
Selective reporting (reporting bias)	Low risk of bias	Study protocol available and all primary, secondary and safety outcomes are reported	Low risk of bias	Study protocol available and all primary, secondary, and safety outcomes are reported	Low risk of bias	Study protocol available and all primary, secondary, and safety outcomes are reported
Other sources of bias	Low risk of bias	The study appears to be free of other sources of bias	Low risk of bias	The study appears to be free of other sources of bias	Low risk of bias	The study appears to be free of other sources of bias
